# Design and Simulation of a Magnetization Drive Coil Based on the Helmholtz Principle and an Experimental Study

**DOI:** 10.3390/mi14010152

**Published:** 2023-01-06

**Authors:** Changlong Zhao, Qiyin Lv, Junbao Yang, Ming Li, Qinxiang Zhao, Hongnan Ma, Xiaoyu Jia

**Affiliations:** College of Machinery and Vehicle Engineering, Changchun University, Changchun 130022, China

**Keywords:** magnetic field generator, Helmholtz coil, 4D printing, magnetic induction

## Abstract

In order to realize the magnetization of hydrogel mixed with NdFeB powder, a magnetization drive coil based on a Helmholtz coil is designed in this paper. The 3D model of the magnetic field system is drawn by the Maxwell software 3D module, and the influence of different factors on the magnetic induction intensity is analyzed to obtain the optimized structure of the magnetization drive coil; then, the central magnetic induction intensity and magnetic induction line distribution density of the magnetization drive coil and Helmholtz coil are compared to verify the reliability of the structure optimization. The results show that the central magnetic induction intensity is the highest when the distance between the auxiliary coils is 70 mm, the central magnetic induction intensity of the magnetized drive coil is significantly higher than that of the Helmholtz coil when the number of turns is the same, and the central magnetic induction intensity of the optimized magnetized drive coil can reach 1.37 T with a more uniform and dense magnetic induction line distribution. After building the magnetization drive coil, the magnetic induction intensity of the center of the magnetization drive coil can reach 1.34 T by a handheld digital Gauss meter test, and the error is no more than 2% with the simulation result. This design approach provides a reference for the structural design and operating characteristics analysis of magnetized drive coils and shortens the design cost and development cycle of magnetized drive coils.

## 1. Introduction

4D printing technology is based on 3D printing technology; after replacing general printing materials with composite materials mixed with smart materials, smart static structures are printed with 3D printers, and by applying external stimuli [1] (light, heat, magnetism, electricity, PH), the printed structures will undergo controlled changes in shape and function over time, thus realizing the transformation from static to dynamic structures. Magnetic hydrogel, as a commonly used smart material, is a composite material formed by mixing NdFeB and other magnetic powders into hydrogel [2]. Magnetically responsive smart hydrogel is a kind of smart hydrogel that can respond to the applied magnetic field stimulation, and its main mechanism of action is magnetically driven. The magnetic particles are mainly embedded in the hydrogel network, and the polymer cross-linked network has deformation characteristics, which causes the macroscopic deformation of the magnetically responsive smart hydrogel and at the same time shows various changes such as bending and elongation with the change in the magnetic field, and the network structure of the hydrogel is not destroyed in this deformation [3]. There are many types of ferromagnetic materials, so the following three materials are generally chosen: the first one is rare earth permanent magnet material; the second one is Alnico permanent magnet material; the third one is permanent magnet ferrite; compared with ferrite and NiCo permanent magnet material, NdFeB has better performance parameters and is suitable for the preparation of magnetically responsive hydrogels. Meanwhile, the results of the study on the relationship between magnetic powder performance and particle size show that when the average particle size increases, the fraction of large diameter particles in magnetic powder increases, and the magnetic properties are reduced [4]. Magnetically responsive hydrogels are generally made of NdFeB powder, with an average particle size of 5 μm and a remanent magnetization (Br) of not less than 1.2 T, but NdFeB magnetic powder particles are very small in diameter and do not have magnetic properties themselves, so they need to be magnetized later. The coercivity of NdFeB magnetic powder is high, so the ordinary DC magnet cannot be completed, and the magnetization drive coil needs to be designed to realize the magnetization of hydrogel.

Currently, magnetization drive coils mainly use permanent magnets or energized coils. Although permanent magnets are easy to purchase and are of low cost, the magnetic field that is generated is not uniform [5,6], and the magnetic induction intensity distribution is not controllable; energized coils are mainly divided into Helmholtz coils and energized solenoids [7,8], but the radial magnetic field distribution of energized solenoids is not uniform, and Helmholtz coils can not only produce a large range of a uniform magnetic field near the midpoint of the common axis but are also simple to produce and better meet the design. In recent years, researchers have studied the magnetic induction strength and magnetic field distribution of Helmholtz coils, and Guo et al. [9] proposed and designed a Helmholtz coil-based drive system. Anna et al. [10] designed a cost-effective compensation system based on a square Helmholtz coil and successfully used it for preliminary measurements of zero-field OPM (optically pumped magnetometers). Ramos et al. [11,12,13,14] analyzed the uniformity of the volume away from the central axis and considered varying the spacing of different Helmholtz coils to expand the uniform field region. Panthinuan et al. [15,16,17], on the other hand, designed different structures to improve the uniformity of the magnetic field. Chen et al. [18] designed an electromagnetic generator consisting of a pair of square coils with the same number of turns, side length, height, and thickness based on the operating principle of Helmholtz coils. The shape of the single coil is 640 mm in length and width, 35 mm in thickness, and 42 mm in height. The magnetic field generated by this generator is simulated in the paper using Maxwell EM simulation software, and the central magnetic induction intensity can reach 14 mT when 80 A current is applied in the experiment. Torres Osorio J et al. [19] designed an open-source application MFV (Magnetic Field Visualizer) for analyzing the magnetic field distribution generated by a circular coil system. However, in practice, large magnetic field generators and current generators generate and consume a lot of energy and costs. Mohammad et al. [20] proposed a high-efficiency, high-power passive rectifier for wireless power transmission applications. A commutation capacitor is added to the input side of the rectifier to improve its performance. The output current was changed from the discontinuous to continuous conduction mode with a maximum efficiency of 94.0%. Different from previous studies. Muhammad Saqib et al. [21] designed and developed a 280 mm diameter Helmholtz coil, which produces a uniform magnetic field of 32 mT. The coil is mainly used to measure different parameters of the coil and to reduce the time of EMC (Electro Magnetic Compatibility) testing. Different from the previous studies, this paper focuses on the structural and energy-saving aspects to improve the central magnetic induction strength of the magnetizing drive coil and reduce the coil size at the same time. Based on the characteristics of the Helmholtz coil structure, a new small-sized magnetization drive coil is proposed in this paper to obtain a larger central magnetic induction strength by introducing an auxiliary coil. Using a combination of simulations and experiments, the effect of different parameters on the central magnetic induction strength of the new magnetization drive coil is analyzed by Maxwell simulation software before building the magnetization drive coil in order to obtain the best structure and reduce the production cost. Finally, the magnetic induction strength of the magnetized drive coil was compared with the simulation results to verify the reliability of the design.

## 2. Methods

### 2.1. Mathematical Model of a Magnetic Field System

**Figure 1:**
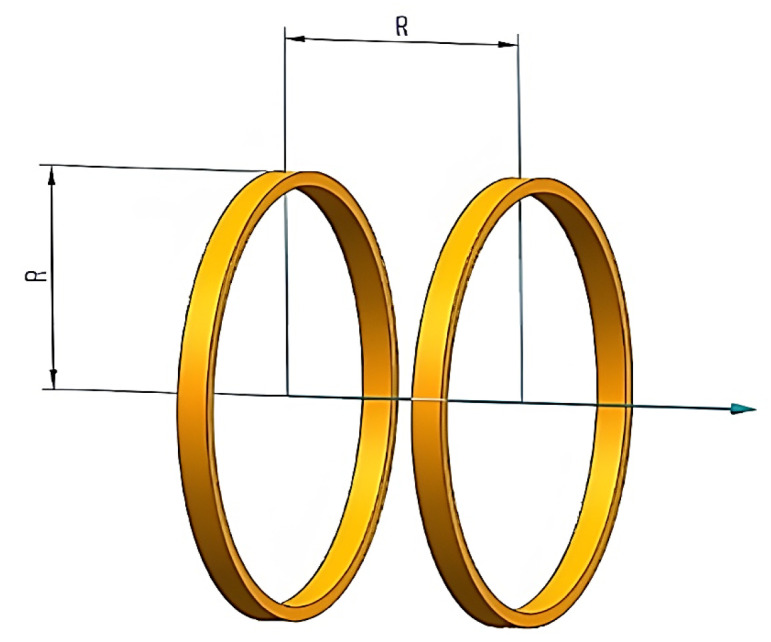
Standard Helmholtz coil structure.

Helmholtz coils consist of a pair of circular single coils with the same number of turns, radius, and winding thickness, which are coaxially placed in parallel [22,23], which can produce a more uniform magnetic field near the midpoint of the axis of the two coils, and the left and right coils are connected in series with the same input current, which will produce a large range of a uniform magnetic field in its center and can be superimposed in space. The uniform magnetic field area will vary proportionally with the radius, so it is often used as a magnetic field generator. The standard Helmholtz coil structure is shown in Figure 1.

The formula for calculating the magnetic induction intensity on the axis of the Helmholtz coil is shown in Equation (1).

(1) $$B\left(x\right)=\frac{1}{2}\mu_{0}NIR^{2}\left\{{\left[R^{2}+{\left(\frac{R}{2}+x\right)}^{2}\right]}^{-\frac{3}{2}}+{\left[R^{2}+{\left(\frac{R}{2}-x\right)}^{2}\right]}^{-\frac{3}{2}}\right\}$$

In Equation (1), *R* is the radius of the coil; *N* is the number of turns of the coil; *I* is the current; $\mu_{0}$ is the magnetic permeability in vacuum (the magnetic permeability in air can be approximated as $\mu_{0}$), $\mu_{0}=4\pi \times 10^{-7}$; x is the distance of any point on the axis from the midpoint of the two coils [19,20].

Using Equation (1), the magnetic field at any point on the axis between the two coils can be calculated. When x=0, the magnetic induction at the midpoint of the two coils on the axis is shown in Equation (2):

(2) $$B\left(x\right)=\frac{1}{2}\mu_{0}NIR^{2}\times 2{\left(\frac{5R^{2}}{4}\right)}^{-\frac{3}{2}}\approx 0.7155\frac{\mu_{0}NI}{R}$$

Finding the first-order derivative of $B\left(x\right)$ shows that $B^{\prime }\left(0\right)=0$. To find the second-order derivative of $B\left(x\right)$:

(3) $$\frac{d^{2}B\left(x\right)}{ds^{2}}=\frac{3}{2}\mu_{0}NIR^{3}\left(\frac{4{\left(\frac{R}{2}+x\right)}^{2}-R^{2}}{{\left[R^{2}+{\left(\frac{R}{2}+x\right)}^{2}\right]}^{\frac{7}{2}}}+\frac{4{\left(\frac{R}{2}-x\right)}^{2}-R^{2}}{{\left[R^{2}+{\left(\frac{R}{2}-x\right)}^{2}\right]}^{\frac{7}{2}}}\right)$$

When x=0, $B^{\prime \prime }\left(0\right)=0$. For the third-order and higher derivatives of $B\left(x\right)$, the value of the derivative at x=0 is the constant 0. It can be proved that $B\left(x\right)$ is homogeneous at x=0.

From Equations (2) and (3), the magnitude of the magnetic field on the axis can be expressed as:

(4) $$B\left(x\right)=B\left(0\right)\left[1-\frac{144}{125}{\left(\frac{x}{R}\right)}^{4}\right]$$

The deviation of the magnetic induction strength from any point on the axis and the origin is:

(5) $$\varepsilon =\frac{B\left(x\right)-B\left(0\right)}{B\left(0\right)}=-\frac{144}{125}{\left(\frac{x}{R}\right)}^{4}$$

From Equation (5), the acquisition of a uniform magnetic field on the coil axis is related to several elements. It is most important to ensure that the magnetic field deviation value is within the range of ±0.1. In order to reach this deviation range, the magnetic field within this range should be calculated, showing the magnetic field deviation value ε≤1 of a circle of 0.3*R*, including the central axis of the circle. Thus, the acquisition of a uniform magnetic field can be obtained by a uniaxial Helmholtz coil.

### 2.2. Structural Design and Simulation Setting

Maxwell 3D is a high-performance 3D electromagnetic design software with precision-driven adaptive dissection technology and a powerful post-processor. The overall characteristics of a motor, bus, transformer, coil, and other electromagnetic components can be analyzed. Graphical results such as the *B* and *H* distribution and the temperature distribution of the analyzed magnets, motor, or magnetized drive coil can also be obtained. In this paper, Maxwell electromagnetic finite element simulation software is used to analyze the effect of additional coils, different coil distances, and different numbers of turns on the central magnetic induction intensity and to determine the optimal parameters of the magnetized drive coil.

#### 2.2.1. Helmholtz Coil Type Selection

The mathematical model analysis is mainly based on the circular Helmholtz coil model, but the Helmholtz coil commonly used in practical applications is divided into two kinds: circular and rectangle. Therefore, before establishing the magnetized drive coil, the central magnetic induction strength of the rectangular Helmholtz coil should be analyzed, and the optimal coil type should be chosen by comparison.

Let the rectangular Helmholtz coil side length be *2a*, *2b*; the magnetic induction *B_zp_* at any point *P (0, 0, Z)* on the center axis of a single coil can be expressed by Equation (6):

(6) $$B_{ZP}=\frac{\mu_{0}NI}{\pi }\left[\frac{ab}{\sqrt{a^{2}+b^{2}+Z^{2}}}\left(\frac{1}{a^{2}+Z^{2}}+\frac{1}{b^{2}+Z^{2}}\right)\right]$$

Let *Z = 0* to obtain the magnetic induction intensity *B_p_* at the center of a single coil plane, expressed by Equation (7):

(7) $$B_{P}=\frac{\mu_{0}NI\sqrt{a^{2}+b^{2}}}{\pi ab}$$

If the rectangular coil is a square coil, then Equation (7) can be reduced to:

(8) $$B_{P}=\frac{\sqrt{2}\mu_{0}NI}{\pi a}$$

Based on the above equation, for the Helmholtz coil composed of two square coils with a side length *2a* and distance *a*, set the middle of the two square coils as the zero point of the coordinate system, and the magnetic induction strength of the central point can be indicated by Equation (9):

(9) $$B_{P}={\frac{4a^{2}NI}{\pi \left[a^{2}+{\left(\frac{a}{2}\right)}^{2}\right]\sqrt{2a^{2}+\left(\frac{a}{2}\right)2}}}^{}=\frac{32NI}{15\pi 5}\approx \frac{0.6791\mu_{0}NI}{a}$$

In Equations (6)–(9), *a* and *b* are the rectangular Helmholtz coil side lengths; *N* is the number of turns of the coil; *I* is the current; $\mu_{0}$ is the magnetic permeability in vacuum (the magnetic permeability in air can be approximated as $\mu_{0}$), $\mu_{0}=4\pi \times 10^{-7}$.

According to Equation (2), the central magnetic field of the circular Helmholtz coil is expressed as follows: $B\left(x\right)\approx 0.7155\frac{\mu_{0}NI}{R}$

When the circular Helmholtz coil diameter is equal to the square Helmholtz coil side length (*2R = 2a*), the circular Helmholtz coil is greater than the square Helmholtz coil.

**Figure 2:**
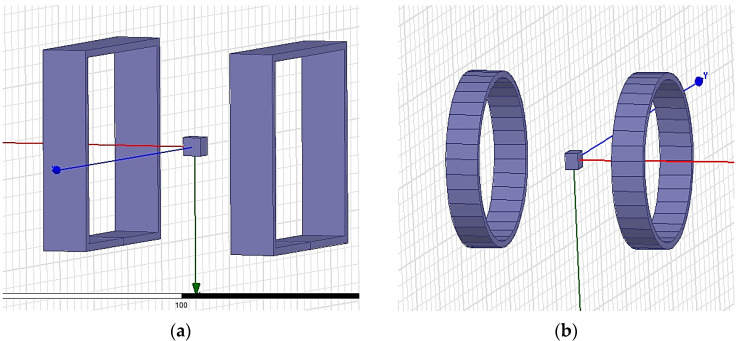
3D model of different coils in the software: (**a**) Square coils; (**b**) Round coils.

In the simulation of the magnetic induction intensity of two coil centers using Maxwell software, the square coil side length is 2*a*, the radius of the round coil is *R*, 2*R =* 2*a =* 110 mm, the height of a single coil is set to 25 mm, and a cube of the size 10 mm *×* 10 mm *×* 10 mm is placed at the center to facilitate the observation of its central magnetic induction intensity. The simplified modeling of the two coils in the software is shown in Figure 2.

The simulation parameters are set as shown in Table 1.

**Figure 3:**
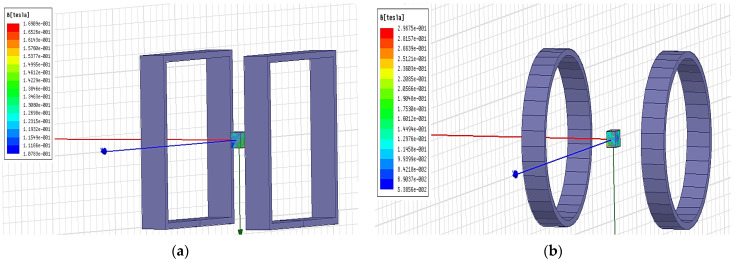
Cloud of magnetic induction intensity at the center of different types of coils: (**a**) Square coils; (**b**) Round coils.

**Figure 4:**
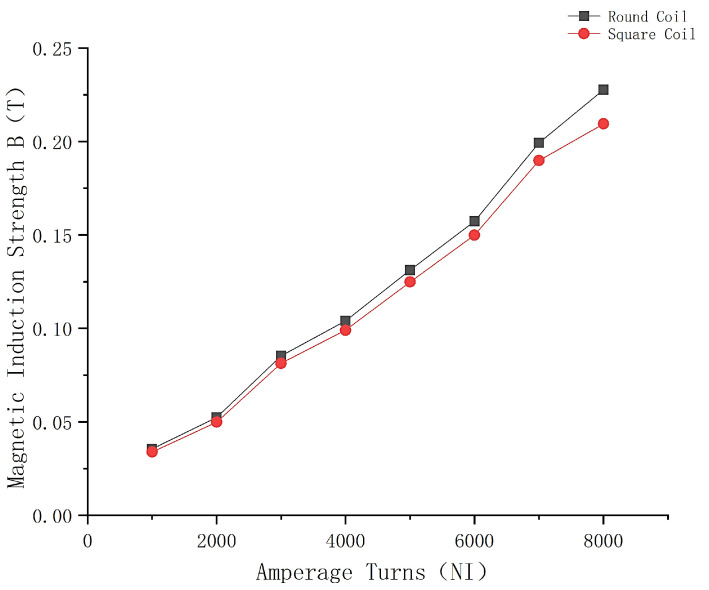
Comparison of the variation curves of the magnetic induction intensity at the center of different types of coils.

The magnitude of the central magnetic induction of the square coil and the circular coil is observed by simulation under the condition of the same number of ampere-turns, and the cloud plot of the central magnetic induction of the two coils is shown in Figure 3. The comparison graph of the central magnetic induction curves of the two coils is shown in Figure 4.

The comparison of the curves in Figure 4 shows that the magnetic induction intensity at the center of the circular Helmholtz coil is greater under the condition of the same number of ampere-turns, which is the same as the result derived from the equation, so the circular Helmholtz coil is chosen for the magnetization drive coil model.

#### 2.2.2. Geometric Model of a Magnetized Drive Coil

For the magnetic field system central magnetic induction strength problem, standard Helmholtz coils are often used in magnetic field generators, but the range of the uniform magnetic field generated is much smaller than its own volume, making the effective magnetic field usage rate low. Moreover, according to the material properties of NdFeB powder, the designed magnetic field-generating device needs to generate a magnetic induction strength of not less than 1.2 T. In practice, the central magnetic induction strength of the Helmholtz coil magnetic field-generating device is around several hundred gausses, and the large-sized magnetic field generator has high production costs and high electrical energy consumption; the central magnetic induction strength of the small-sized magnetic field generator is mostly lower than 0.5 mT. Therefore, the magnetization drive coils with good performance are designed to meet the design requirements.

It is not a reasonable design idea to change only the size of the coil in order to meet the design requirements. Therefore, this paper focuses on optimizing the internal structure of the coil from the perspective of the overall coil structure by adding a pair of auxiliary coils on both sides of the small-sized Helmholtz coil to compensate for the magnetic induction intensity at the center as well as away from the center. By this method of adding auxiliary coils, it is possible to achieve the design requirement of a magnetic induction of not less than 1.2 T and at the same time reduce the coil production cost.

The radius of the auxiliary coil, the distance size of the auxiliary coil, and the ampere-turn ratio of the main and auxiliary coils are the three main parameters affecting the magnetic induction intensity at the center of the proposed new magnetic field driving coil. The number of turns of the main coil is initially set to 6000, and for the magnetic field-generating device proposed in this paper, the ratio of the number of turns of the main and auxiliary coils is set to 1, considering the spatial symmetry.

Considering the space inside the magnetic field generator required for the experiment and that the effective magnetization area of the magnetization drive coil should be not less than ∅ 20 × 20 mm, a pair of circular coils with the same number of turns, inner diameter, outer diameter, and height were used as auxiliary coils, placed parallel to the common axis.

**Figure 5:**
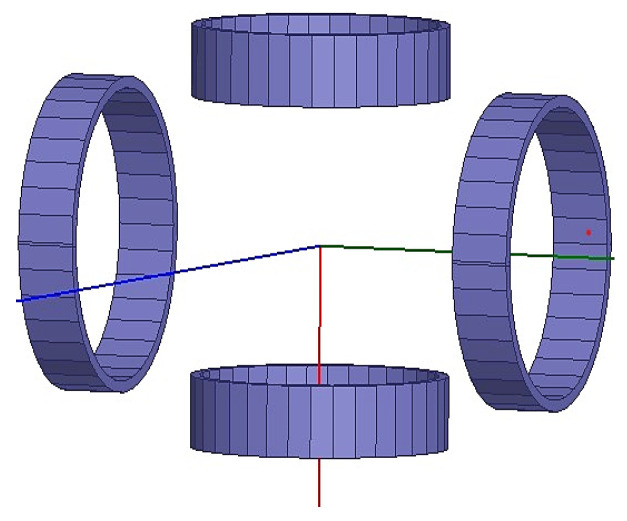
Simplified modeling diagram of the new magnetic field-driven coil.

(1) For the main coil, the outer diameter and inner diameter are 70 mm and 66 mm, respectively; for the auxiliary coil, the outer diameter and inner diameter are 55 mm and 51 mm, respectively;
(2) The height dimension of each coil is 25 mm;
(3) The distance between the auxiliary coils is set to 80 mm and placed symmetrically on the left and right sides of the main coils. The simplified 3D modeling of the designed magnetic field drive coils in Maxwell software is shown in Figure 5.

#### 2.2.3. Model Meshing and the Associated Parameter Settings

**Figure 6:**
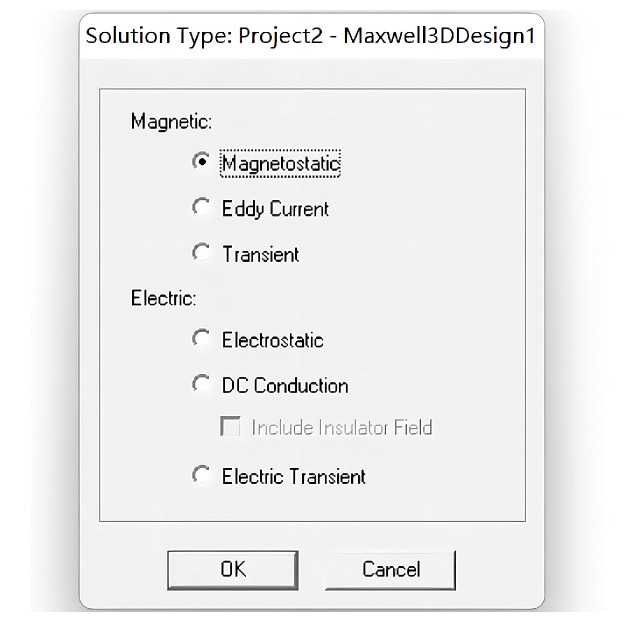
Solution type selection diagram.

The magnetostatic static magnetic field solver was analyzed here, as shown in Figure 6.

**Figure 7:**
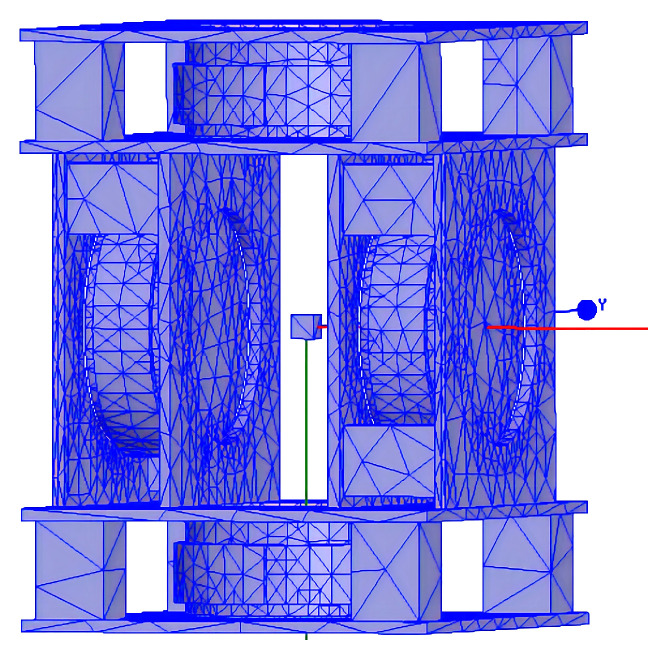
Mesh division diagram of the magnetic field generator.

The side length of the main coil fixing plate for fixing is 150 mm × 150 mm, and the thickness is 5 mm. In order to facilitate simulation calculations and reduce the simulation time, the simplified model needs to be drawn in the software. After the simplified model is drawn, it needs to be meshed. Maxwell 3D can automatically divide the mesh according to the model, so there is no need to set the parameters separately. The mesh division diagram is shown in Figure 7.

After the grid division is completed, the calculation area is created, the excitation source is set, the material is set, etc. The settings of the relevant simulation parameters are shown in Table 2.

## 3. Results and Analysis

### 3.1. Simulation Results and Analysis

In the simulation analysis, there are two main parameters analyzed for the drive coil, which are the number of ampere-turns of the main coil and the auxiliary coil and the distance between the two auxiliary coils. The variation in the magnetic induction intensity in the center of the magnetized drive coil is obtained by changing different parameters, which provides a reference for the design of the magnetized drive coil structure. Meanwhile, a cube of the size 10 mm × 10 mm × 10 mm is placed at the center of this magnetization drive coil to facilitate the observation of its central magnetic induction intensity.

(1) Simulation analysis of the effect of auxiliary coil distance on the central magnetic induction intensity

**Figure 8:**
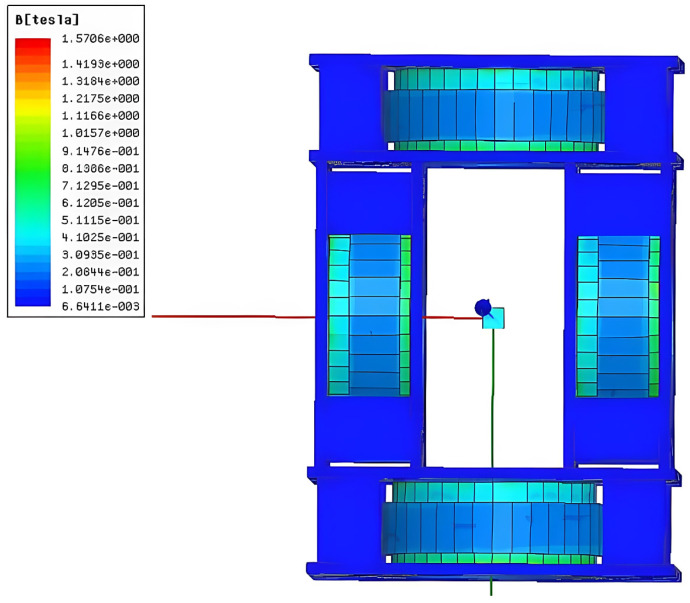
Cloud map of magnetic induction intensity at 70 mm.

**Figure 9:**
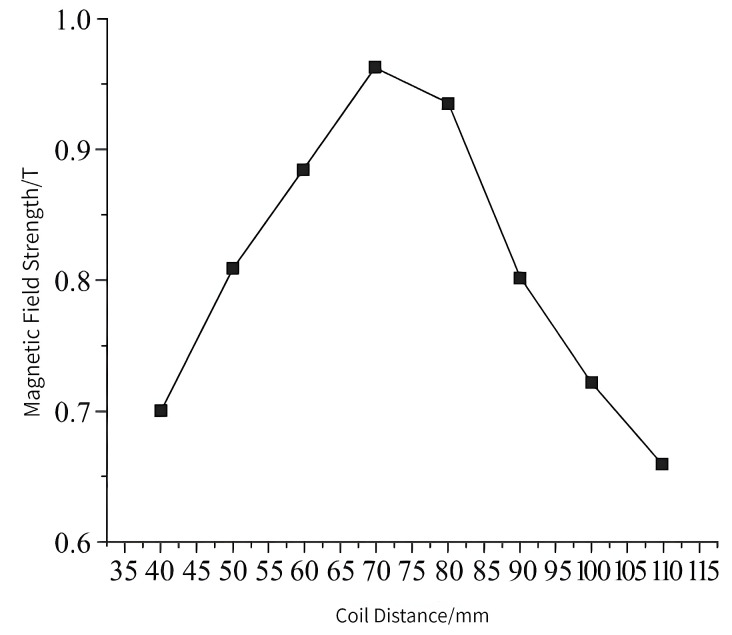
Changes in central magnetic induction intensity with coil distance.

In the magnetization drive coil, the distance between the auxiliary coils is inversely related to the central magnetic induction intensity, i.e., the further the distance between the auxiliary coils, the smaller the central magnetic induction intensity, but when the distance between the two coils is too small, it interferes with the magnetic field generated by the main coil itself. Therefore, it is necessary to optimize the parameter of the distance of the auxiliary coil. The distance of the auxiliary coil is 40–110 mm, and the number of turns is 6000. When the distance of the auxiliary coil is 70 mm, the cloud diagram of the central magnetic induction is shown in Figure 8, and the variation in the central magnetic induction with the distance of the auxiliary coil is shown in Figure 9.

From the simulation results, it can be seen that the central magnetic induction intensity changes with the change in the distance of the auxiliary coil, and the magnetic induction intensity is maximum when the distance of the auxiliary coil is 70 mm. Therefore, 70 mm is selected as the optimal distance of the auxiliary coil.

(2) The simulation analysis of the influence of Ann turns on the center induction of the Helmholtz coil and magnetized drive coil

**Figure 10:**
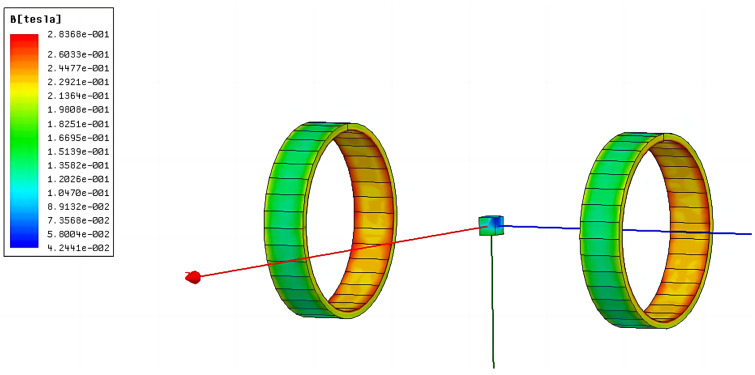
Cloud diagram of the Helmholtz coil magnetic induction intensity.

**Figure 11:**
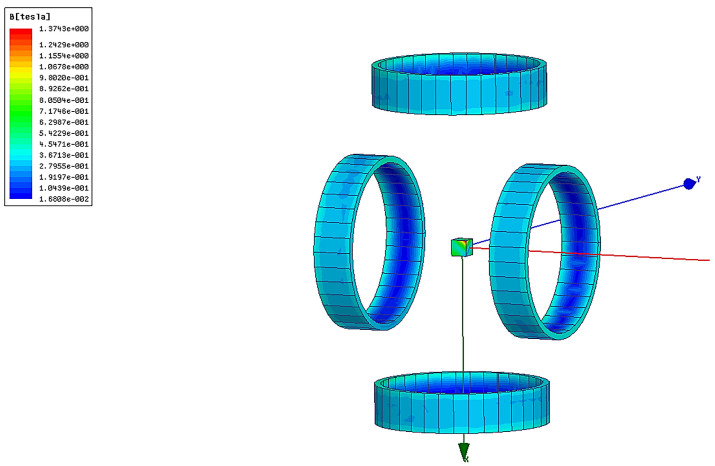
Cloud diagram of the magnetic induction strength of the magnetized drive coil.

**Figure 12:**
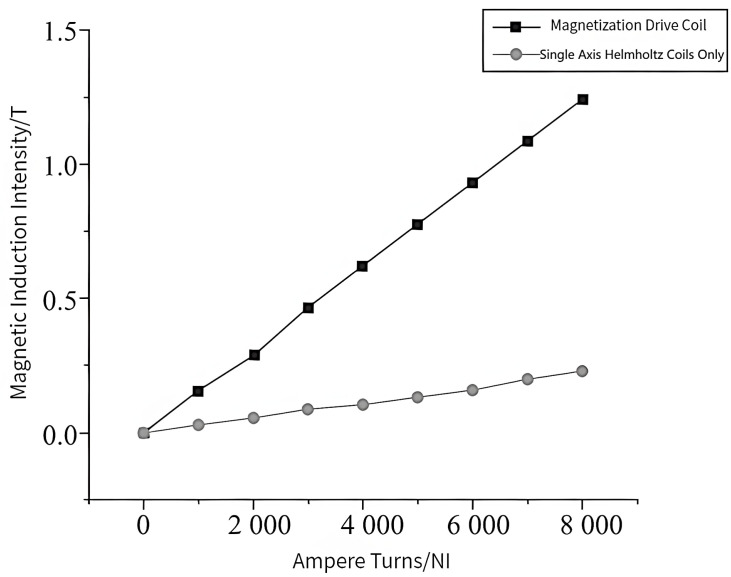
Comparison of the magnetic induction intensity characteristic curves of magnetized drive coils and Single-Axis Helmholtz Coils Only.

According to the design requirements, the central magnetic induction strength of the magnetization drive coil needs to be higher than 1.2 *T*. After selecting the optimal auxiliary coil distance, the simulation of the number of turns is also required to analyze its effect on the central magnetic induction strength of the magnetization drive coil and select the optimal parameters. The magnetic induction intensity clouds of the Helmholtz coil and magnetization drive coil are shown in Figure 10 and Figure 11. A comparison of the magnetic induction intensity characteristic curves generated at the midpoint of the center axis of the magnetized drive coil and the Single-Axis Helmholtz Coils Only is shown in Figure 12.

The central magnetic induction of both the magnetized drive coil and the Helmholtz coil is proportional to the number of ampere-turns; the central magnetic induction increases with the number of ampere-turns. Figure 12 shows that the central magnetic induction of the magnetized drive coil is significantly higher than that of the Helmholtz coil. The central magnetic induction of the magnetized drive coil can reach 1.37 T at the ampere-turn number of 8000, which can meet the design requirements.

(3) Distribution of Helmholtz coils and magnetized drive coils

**Figure 13:**
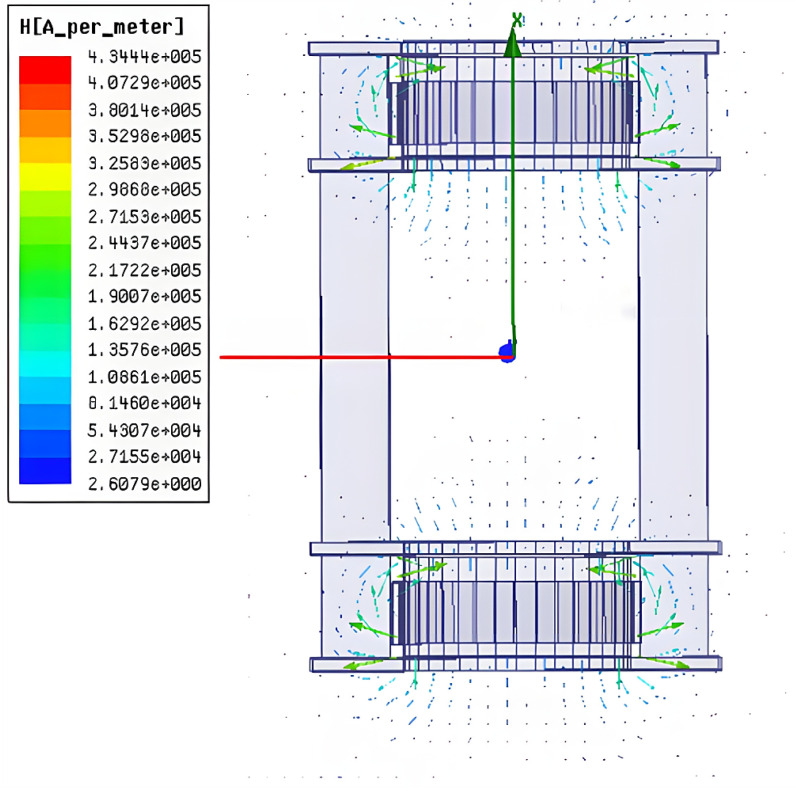
Helmholtz coil X-Y plane magnetic induction line distribution.

**Figure 14:**
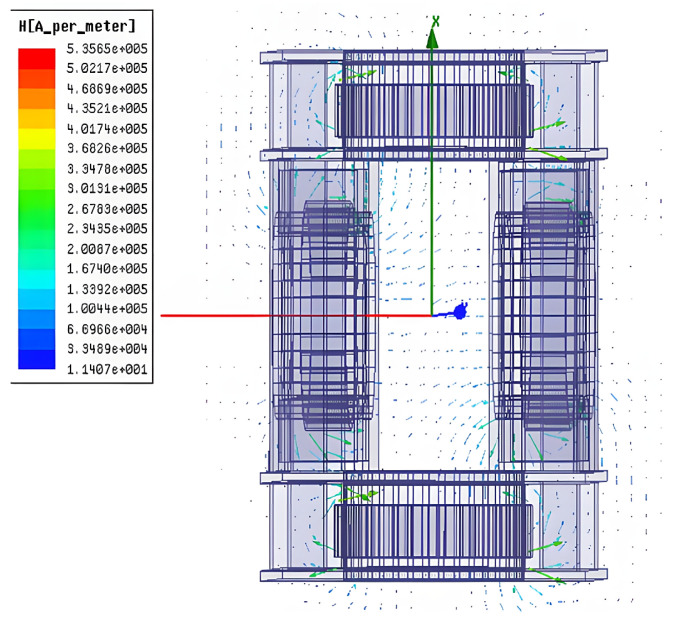
Magnetization drive coil X-Y plane magnetic induction line distribution.

The distribution of magnetic induction lines in the X-Y plane of the Helmholtz coil is shown in Figure 13, and the distribution of magnetic induction lines in the X-Y plane of the magnetization drive coil is shown in Figure 14. By comparing Figure 13 and Figure 14, it can be seen that the magnetization drive coil with the addition of the auxiliary coil has a more uniform and dense inductance distribution.

After optimizing the distance of the auxiliary coil and the number of turns of the main and auxiliary coils in the magnetization drive coil, it can be seen that when the distance of the auxiliary coil is 70 mm and the number of turns of the main and auxiliary coils is 8000, the magnetic induction intensity in the center of the magnetization drive coil can reach 1.37 T. After increasing the auxiliary coil, the magnetic inductance distribution of the magnetization drive coil is more dense and uniform. The above analysis shows that the magnetization drive coil design is reasonable and can meet the design requirements.

(4) Temperature characteristics analysis

**Figure 15:**
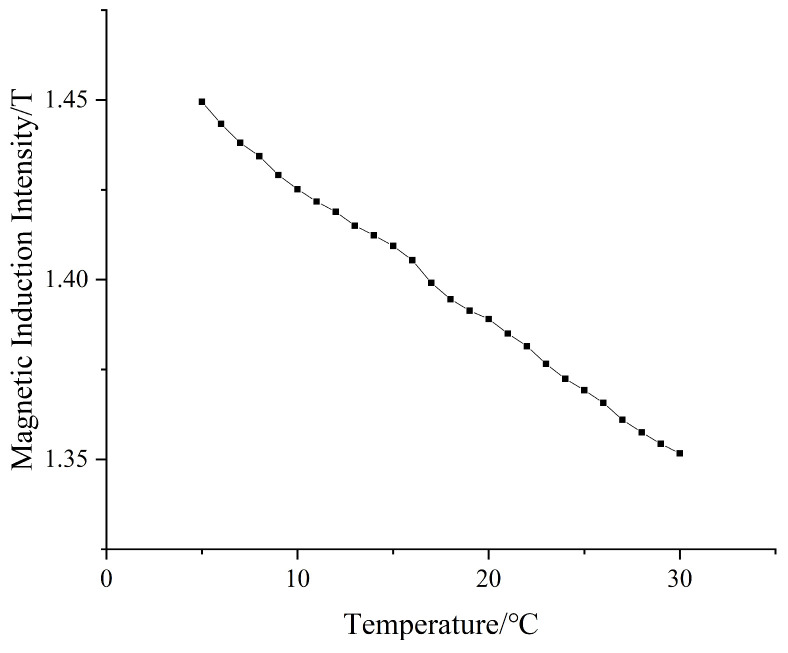
Temperature characteristic curve.

Since the NdFeB magnetic material doped in the magnetically responsive hydrogel has a high temperature coefficient, the change in temperature will change the magnetic property of NdFeB, so we need to study the effect of the operating temperature on the system performance. In order to investigate the temperature characteristics of the magnetized drive coil model, 25 sets of simulations are made in Maxwell by gradually increasing the operating temperature from 5 °C to 30 °C, with an interval of 1 °C for each set of simulations. By comparing the central magnetic induction of the magnetized drive coil at different temperatures, the effect of the change in the operating temperature on the system performance is analyzed. The temperature coefficient of the NdFeB residual magnetic induction strength Br is set to −0.12%, and the coercivity temperature coefficient is set to −0.70% in the simulation. The central magnetic induction intensity at different operating temperatures is shown in Figure 15.

The temperature characteristic curve shows that the central magnetic induction strength decreases with the increase in the operating temperature. In this paper, the working temperature set in the simulation software Maxwell is 25 °C, that is, the magnetization drive coil is designed and optimized under the condition of a working temperature of 25 °C. In actual operation, the magnetization drive coil will be placed to work under the condition of room temperature (23–25 °C), and it can be seen from the temperature characteristic curve that the central magnetic induction intensity is greater than 1.355 T when the temperature is lower than 30 °C, which meets the experimental conditions.

The temperature characteristic curve of the magnetization drive coil shows that there is a certain function relationship between the magnetic induction strength *B* and operating temperature *T*. However, due to the hysteresis and saturation characteristics of magnetic materials, an empirical model is needed to express the relationship between the magnetic induction strength and temperature more accurately. In this paper, the empirical model is established by the temperature characteristic of NdFeB remanent magnetism, and the function relationship is shown in Equation (10):

(10) $$B_{T}=B_{T0}\left[1+\mu \left(T-T_{0}\right)\right]$$

where *T* is the working temperature, *B_T_* is the central magnetic induction of the magnetized drive coil at a specific working temperature, *T*_0_ is the room temperature, *B_T_*_0_ is the central magnetic induction at room temperature, and μ is the ambient temperature coefficient; according to the simulation data and parameter settings, Equation (10) can finally be transformed into Equation (11):

(11) $$B_{T}=B_{T0}\left[1-0.0032\left(T-T_{0}\right)\right]$$

### 3.2. Coil Physical Construction and Analysis

In practice, magnetic field coils are usually made up of a combination of coil architecture, insulation, enameled wire, and other ancillary components. When the coil is fed with current, it will heat up because of its own resistance. The heat of the coil will affect the performance of the magnetic field and shorten its service life, so the following factors are generally considered when designing the coil:

(1) Designed with the magnetic potential to meet the magnetic field drive requirements.
(2) Temperature rises at stable operation < limit temperature rises of insulation material.

**Figure 16:**
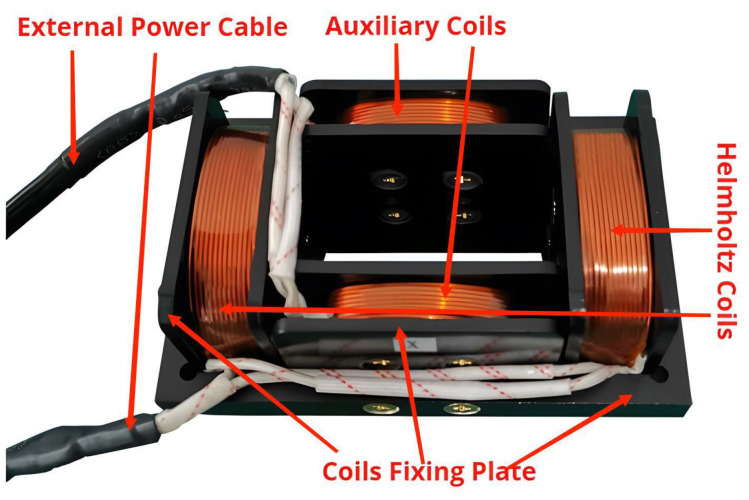
The physical diagram of the magnetized drive coils.

The skeleton of the unit coil of this system is aluminum profile, industrial plastic, and PI polyimide film, which can play the role of electromagnetic isolation and has a certain temperature resistance. The coil is wound with enameled wire, and each layer of the enameled wire is fixed with double-sided adhesive and can play the role of isolation. The physical diagram of the magnetized drive coils is shown in Figure 16.

**Figure 17:**
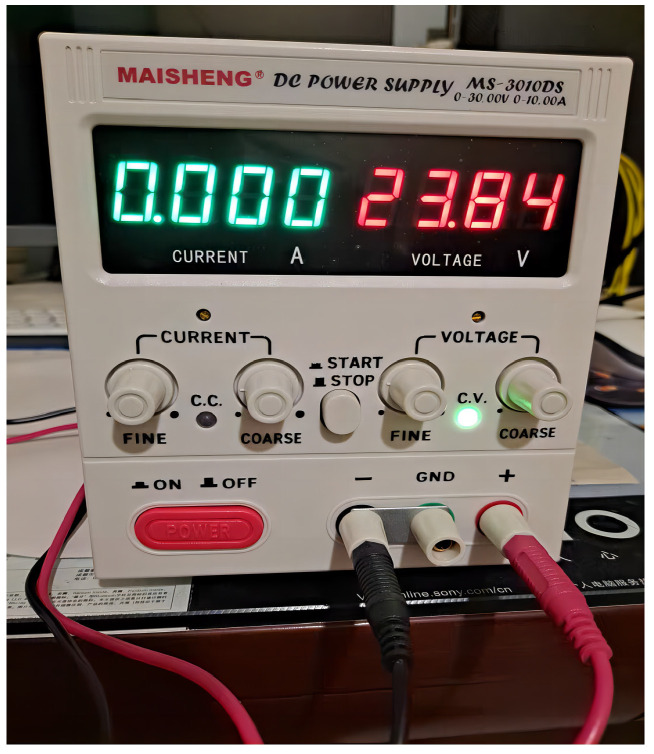
M S DC Power Supply.

The magnetization drive coil power supply in this paper uses a DC power supply of the MS series. This DC power supply is shown in Figure 17. This instrument is highly stable, with less fluctuation and continuously adjustable voltage changes, and can be used as a stable voltage and current source, which is suitable as a power supply for magnetization drive coils. The biggest advantage of the MS series power supply is that it can be connected in series or parallel to change the maximum voltage and maximum current it can provide, and this use allows it to meet most of the MS series power supply. The output voltage range is 0–50 V and the output current range is 0–10 *A*. The overall experimental test shows that this MS series DC power supply (MAISHENG, Suzhou, China) can meet the demand of the magnetization drive coil power supply.

**Figure 18:**
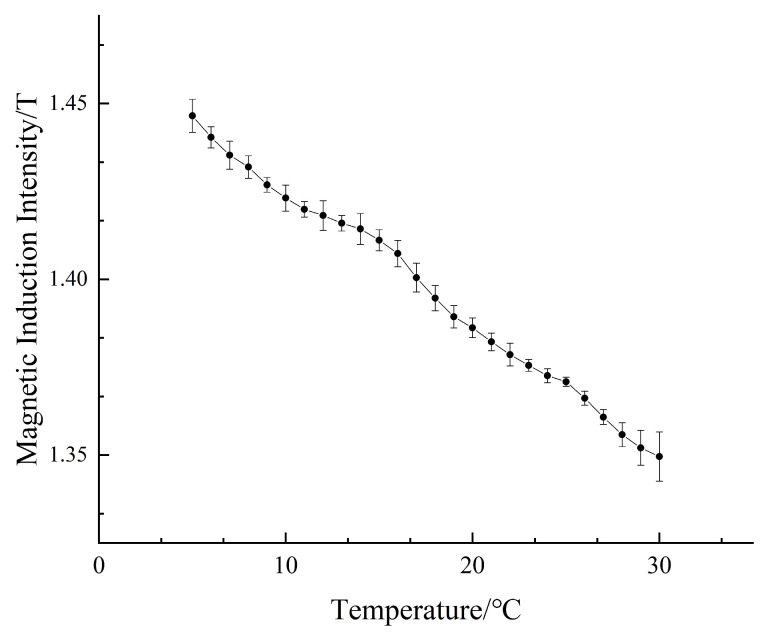
Actual operating temperature characteristic curve of the magnetized drive coil.

The magnetic field generated by the magnetization drive coil is performed by three MS series DC power supplies. The first power supply enables the main coil to generate a uniform magnetic field to magnetize the hydrogel. The second and third power supplies enable the auxiliary coils to generate a gradient magnetic field, which is used to magnetize and deform the magnetic soft robot by varying the current magnitude. Through the experimental test, the effective magnetization area of the magnetization drive coil can reach 25 × 25 mm, the magnetization effect decreases with the increase in hydrogel thickness, and the maximum magnetization thickness can reach 20 mm. In previous studies, the current through the coil ranged from 5 to 100 A, and the magnetic induction intensity produced ranged from a few dozen mT to several hundred mT. Through the handheld digital Gauss meter test, the magnetization drive coil designed in this paper produced a magnetic induction intensity of up to 1.34 T, when the current through the coil ranged from 0 to 10 A, and at the same time, the error with the simulation results did not exceed 2%. The actual operating temperature characteristic curves of the magnetized drive coil at different operating temperatures are obtained by measurement, as shown in Figure 18.

**Figure 19:**
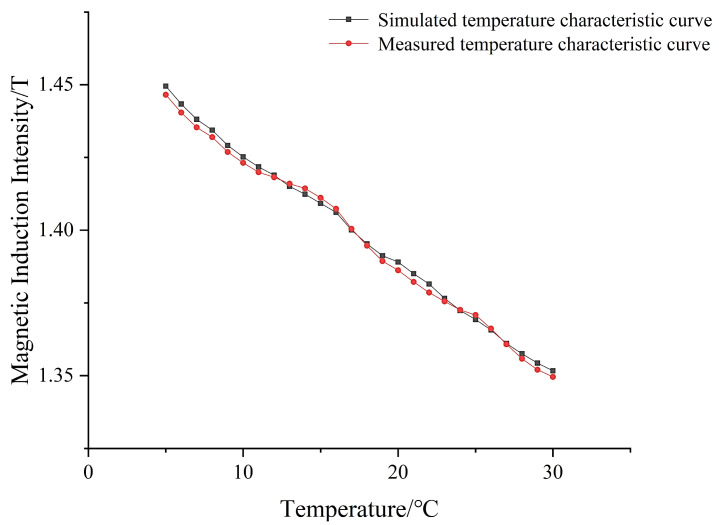
Comparison of the simulated and measured temperature characteristic curves.

The comparison of the actual measured data with the simulated data is shown in Figure 19. From the curves in Figure 19, it can be seen that the simulated data fit better with the actual measured data, which indicates that the empirical model established by the simulation is correct. All the experimental results can prove that the design is reasonable, and the magnetic induction strength of the magnetized drive coil can meet the experimental requirements.

## 4. Conclusions

In this paper, based on the principle of the Helmholtz coil, a new type of magnetization drive coil is obtained by adding a pair of auxiliary coils on both sides of the coil to compensate for the field strength at the center point of the Helmholtz coil and away from the center point. This magnetization drive coil has a small size, high central magnetic induction strength, dense magnetic induction distribution, and low fabrication cost.

First, we simulate the magnetization drive coil model by using the finite element analysis software Maxwell and select the circular Helmholtz coil with a large central magnetic induction as the unit coil of the magnetization drive coil. Then, we build a three-dimensional model, analyze the influence of the distance between the two auxiliary coils on their central magnetic induction, and select the optimal auxiliary coil distance. We change the number of turns to analyze its influence on the central magnetic induction of the Helmholtz coil and the magnetization drive coil. Finally, we compare the optimized magnetization drive coil and Helmholtz coil magnetic induction distribution to prove the reliability and design rationality of the magnetization drive coil.

The experimental results show that 70 mm is the optimal value of the distance of the auxiliary coil; the central magnetic induction intensity is basically positively correlated with the size of the number of turns, the central magnetic induction intensity of the magnetized drive coil is significantly higher than that of the Helmholtz coil after adding the auxiliary coil, and the final optimized magnetized drive coil has a more uniform and dense magnetic induction distribution. When the distance of the auxiliary coil is 70 mm and the number of turns is 8000, the central magnetic induction intensity can reach 1.37 T. After the construction of the magnetization drive coil, through experimental testing, the effective magnetization area of the magnetization drive coil can reach 25 × 25 mm, the magnetization effect decreases with the increase in hydrogel thickness, and the maximum magnetization thickness can reach 20 mm. Compared with previous studies, the magnetized drive coil proposed in this paper is not only smaller in size and less expensive to fabricate but also generates a higher central magnetic induction intensity when the incoming current is 0–10 A. The central magnetic induction of the magnetization drive coil can reach up to 1.34 T by the handheld digital Gauss meter test, which is within 2% error with the simulation results, proving that the design is reasonable and that the central magnetic induction of the magnetization drive coil can meet the experimental requirements. By simulating and analyzing the magnetic field generation device, the designer can change the parameters and choose a reasonable magnetization drive coil structure according to different situations, which shortens the time of developing the magnetization drive coil and reduces the resource consumption at the same time.

Compared with previous studies, although the magnetized drive coil proposed in this paper is smaller in size and cheaper to fabricate and can produce a stronger magnetic induction strength under the same current pass, it still needs to be enhanced and improved. The current source is still controlled manually. Future work can focus on the current source control system and develop an intelligent control system by writing a program to achieve precise control of the magnitude and timing of the incoming current. After the precise control of the current source, we can continue to increase the number of auxiliary coils, study the effect of different placement angles and placement distances on the central magnetic induction intensity, and obtain the magnetic field in different directions by controlling the current into the coils. For the control of the operating temperature, the temperature control module can be designed to ensure the stability of the operating temperature in the future.

## Figures and Tables

**Table 1:** Simulation parameters of the static magnetic field.

Parameter	Setting
Region	100%
Material	Coil: copper
Driving Source	Current source: the number of Ann turns, Direction: Negative
Analysis Setup	Maximum number of iterations: 5 Percent Error: 1% Refinement per Pass: 0.3

**Table 2:** Simulation Parameters of the static magnetic field.

Parameter	Setting
Region	100%
Material	Upper and lower fixed plate, coil fixed column, profile: aluminum Coil: copper
Driving Source	Current source: Ann turns, direction: Negative
Analysis Setup	Maximum number of iterations: 5 Percent Error: 1% Refinement per Pass: 0.3

## Data Availability

Data from this study are available from the corresponding authors upon request. Due to privacy, these data are not publicly available.
